# Body mass trajectory from diagnosis to the end of treatment in a pediatric acute lymphoblastic leukemia cohort

**DOI:** 10.1038/s41598-023-39287-z

**Published:** 2023-08-21

**Authors:** Paula Cristina Galati, Priscilla Roberta Silva Rocha, Nádia Dias Gruezo, Angélica Amorim Amato

**Affiliations:** 1 Laboratory of Molecular Pharmacology, School of Health Sciences, University of Brasilia, Brasilia, Brazil; 2 Children’s Hospital of Brasilia José Alencar, Brasilia, Brazil; 3 Faculty of Ceilândia, University of Brasilia, Brasilia, Brazil

**Keywords:** Endocrinology, Oncology

## Abstract

The advances in pediatric acute lymphoblastic leukemia (ALL) care have substantially increased survival, and the late effects of treatment are a growing concern. Obesity development is frequent following ALL therapy and may significantly contribute to long-term morbidity and mortality. We examined the body mass trajectory of 208 children with ALL, from the diagnosis to the completion of therapy. We found that 7.2% of children were overweight or obese at diagnosis, which increased to 19.7% at the end of induction therapy and 20.8% after completion of treatment. In a multivariable linear regression model, age at ALL diagnosis, the type of chemotherapy regimen, and body mass index (BMI) z-score at diagnosis were significant predictors of BMI z-score after induction therapy, whereas BMI z-score at diagnosis was the only significant predictor of BMI z-score at the completion of treatment. In a subgroup of 120 children, we found no association between nutrition status at diagnosis and the risk of ALL relapse or poorer overall survival. Our findings indicate that weight gain occurs early during ALL therapy and is predicted by weight status at diagnosis. Therefore, nutritional status should be assessed throughout treatment, and weight management interventions should be considered early, particularly for patients with higher weight at diagnosis.

## Introduction

The prevalence of overweight and obesity has increased significantly among children and adolescents in many countries over the last decades^1^ and is currently viewed as a growing pandemic. Excess body weight during childhood has substantial short-term and long-term consequences. Children who are overweight or obese are more likely to develop psychological disorders^2,3^, metabolic abnormalities, and cardiovascular risk factors^3^. Moreover, the overweight status can persist into adulthood^4^ and poses significant morbidity and premature mortality^5,6^.

Various studies indicate that obesity is associated with an increased likelihood of developing multiple types of cancer in adulthood^7,8^. In the pediatric population, the potential role of obesity on cancer development is unclear, but obesity and cancer are still related in other ways. Childhood cancer treatment frequently leads to weight gain that may increase the risk for premature morbidity and mortality among cancer survivors^9^. Moreover, excess body weight at childhood cancer diagnosis may negatively affect treatment outcomes.

Acute lymphoblastic leukemia (ALL) is the most common pediatric malignancy^10^, and findings from several studies indicate that weight status negatively affects its prognosis^11^. Children who are overweight or obese at diagnosis exhibit an increased risk of ALL recurrence and reduced event-free survival rates^11,12^, although data on overall survival rates are more variable^11,13,14^. The mechanisms linking obesity to poorer pediatric ALL outcomes are incompletely understood. Still, there is increasing evidence from preclinical studies indicating that systemic and local adipose tissue abnormalities in obesity favor the survival of leukemic cells^15^.

In addition, several studies have examined the weight patterns in patients with childhood ALL at diagnosis, during, and after treatment. These studies consistently identified a significant increase in the rate of overweight or obesity at all stages of treatment^16–18^. Weight gain can develop throughout ALL treatment due to various factors related to the action of chemotherapeutic agents and an unhealthy environment, such as prolonged exposure to glucocorticoids, hormonal changes, reduced physical activity, and an unhealthy diet^9^. Given the improvement in the survival rates for pediatric ALL^19^, persistent weight increase during treatment is a significant concern. It may place ALL survivors at an increased risk for other chronic health conditions and adversely impact long-term morbidity and mortality outcomes^9,20^.

In this scenario, examining weight patterns during and following ALL treatment to identify the sensitive windows of unhealthy weight gain and the predictors of weight increase is important for developing strategies to prevent treatment-related obesity and its long-term consequences among ALL survivors. In this study, we describe the body mass trajectories of children with ALL before and throughout treatment and investigate its predictors and association with disease relapse at four years.

## Results

### Characteristics of study subjects

**Figure 1:**
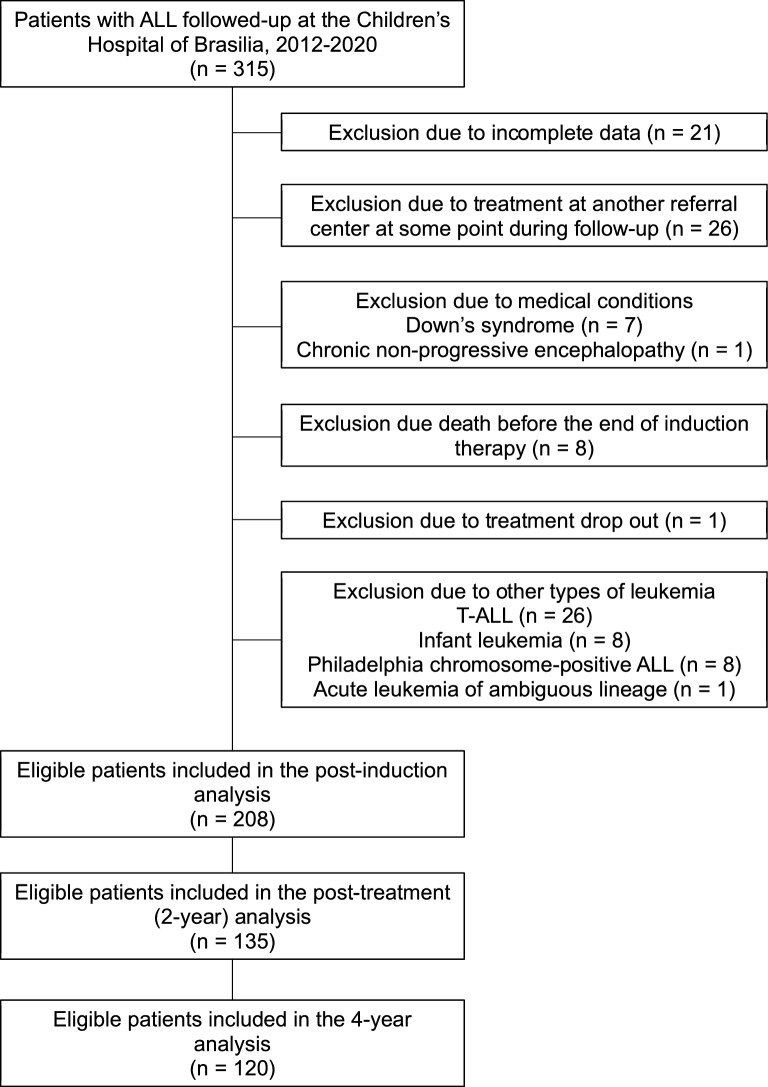
Flow diagram of patient selection. ALL: acute lymphoblastic leukemia.

A total of 315 children were diagnosed with ALL between January 2012 and July 2020. After excluding ineligible patients, 208 were assessed at the time of ALL diagnosis and in post-induction treatment (1-month follow-up), 135 children had completed treatment (2-year follow-up), and 120 children had been followed-up for 4 years (Fig. 1). Their demographic, clinical, and treatment-related characteristics are presented in Table 1. The median age at ALL diagnosis was 4.4 years, and there was a slight male predominance (57%). Most included children were treated according to the GBTLI-93 protocol (69%), were considered standard (42%) or high-risk (39%), and had no CNS disease (96%). Only 7.2% of the participants were overweight (5.8%) or obese (1.4%) at diagnosis. Compared with non-overweight/non-obese children, those who were overweight/obese were significantly older at diagnosis (Table 1). No significant differences in gender, central nervous system (CNS) disease, risk stratification, white blood cell (WBC) count at diagnosis, or chemotherapy protocol were found between the children with overweight/obesity and those who were not overweight or obese at diagnosis.

**Table 1:** Demographic and clinical characteristics of the study participants at diagnosis.

Characteristic	All (n = 208)	BMI category at diagnosis		p-value
		Non-overweight/non-obese (n = 193)	Overweight/obese (n = 15)	
Gender (%)				0.7911*
Female	43	42.5	46.7	
Male	57	57.5	53.3	
Age at diagnosis—years (median, IQR)	4.4 (3–7.8)	2.8 (1.9–4.8)	4.6 (3–8.2)	0.0041**
< 10 years (%)	80	81.3	60	
≥ 10 years (%)	20	18.7	40	
Weight status—BMI z-score (%)				
Underweight (< − 2)	7			
Normal weight (− 1.9 to 0.9)	86			
Overweight (1.0 to 1.9)	4			
Obesity (≥ 2)	3			
CNS disease (%)	4	4.2	0	0.8931*
Cranial irradiation (%)	0	0	0	–
Risk stratification (%)				
Low	19	19.2	20	
Standard	42	41.5	46.7	
High	39	39.3	33.3	
Chemotherapy protocol (%)				> 0.9999*
BFM-95	31	69	66.6	
GBTLI-93	69	31	33.4	
WBC count at diagnosis—× 10^9^/L (%)				
< 10	52.8	52.8	46.6	0.8608*
10–49.9	30	29	40	
50–99.9	8.2	8.8	6.7	
≥ 100	9	9.3	6.7	

*BFM* European Group Berlin-Frankfurt-Munich protocol, *BMI* body mass index, *GBTLI* Brazilian Group for the Treatment of Childhood Leukemia, *IQR* interquartile range, *WBC* white blood cell.

*Fisher’s exact or chi-squared test.

**Mann–Whitney test.

### Body mass index trajectory from diagnosis to treatment completion

**Figure 2:**
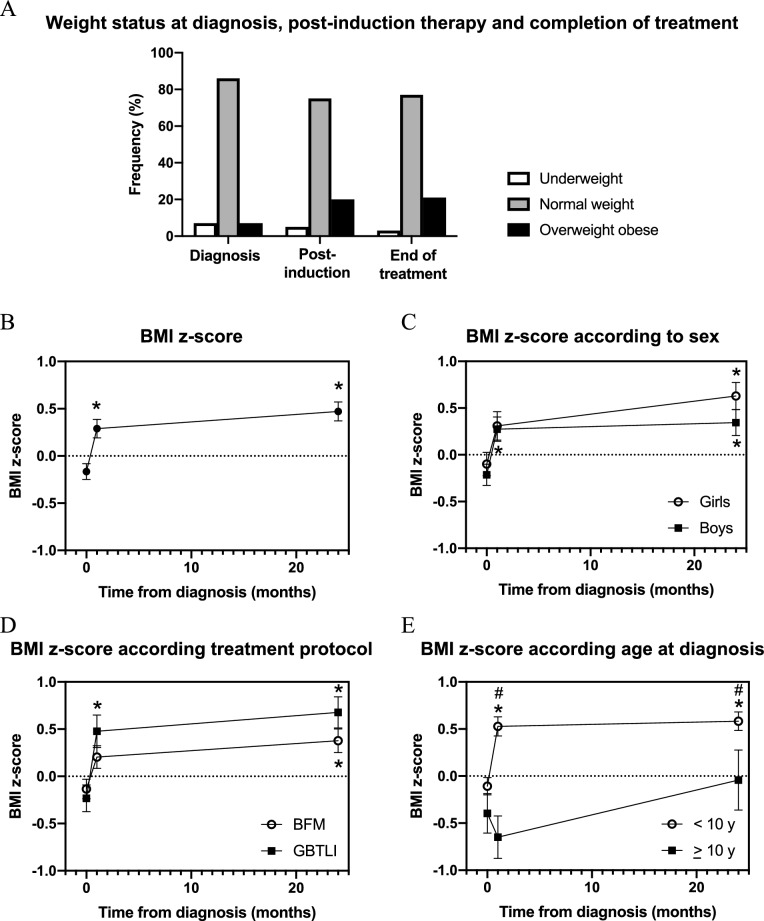
Body mass index z-score trajectory of children with acute lymphocytic leukemia from diagnosis to the end of treatment. (**A**) Nutritional status by body mass index category at diagnosis (n = 208), post-induction (n = 208), and end of treatment (n = 135). Body mass index z-score trajectory from (**B**) all included children (n = 208 at diagnosis, n = 208 at 1 month, and n = 135 at 24 months) and according to (**C**) gender (n = 89 girls and 119 boys at diagnosis and 1 month, and n = 60 girls and 75 boys at 24 months), (**D**) treatment regimen (BFM-95 vs GBTLI), and (**E**) age at diagnosis (n = 166 < 10 years and 42 > 10 years at diagnosis and 1 month, and n = 111 < 10 years and 24 > 10 years at 24 months). *p < 0.05 vs z-score at diagnosis, by analysis of variance followed by Tukey’s multiple comparison test, ^#^p < 0.05 by unpaired t-test (boys vs girls or age at diagnosis < 10 years vs > 10 years). Data presented as mean ± standard error of mean.

Most included children had normal weight at diagnosis (85.6%). Only 7.2% were overweight or obese, and 7.2% were underweight. After induction therapy, there was a significant increase in the frequency of overweight/obesity (19.7%), which persisted at the end of treatment, affecting 20.8% of children (Fig. 2A). Accordingly, the body mass index (BMI) z-score trajectory from diagnosis to end of treatment, at 24 months, indicated a significant increase after induction therapy (1 month), which persisted at the end of treatment (24 months, Fig. 2B).

BMI z-score trajectory was similar when comparing boys with girls (Fig. 2C). Moreover, the mean BMI z-score of children treated with the Brazilian Group for the Treatment of Childhood Leukemia (GBTLI) chemotherapy protocol was higher than that of children treated with the European Group Berlin-Frankfurt-Munich 95 (BFM-95) protocol both after induction therapy and after the completion of treatment, but this difference was not statistically different (Fig. 2D). Children treated with the GBTLI protocol had a higher median BMI z-score at diagnosis (+ 0.02, interquartile range − 0.53 to + 0.70) than those treated with the BFM-95 protocol (− 0.18, interquartile range − 1.08 to + 0.59), although the difference was not significant (p = 0.18, Mann–Whitney test). There was no difference in age at diagnosis when comparing children treated with either protocol (median 4.25 years for GBTLI and 4.83 years for BFM-95, p = 0.46, Mann–Whitney test).

BMI z-score trajectory was significantly different when comparing children with ALL diagnosis before and after the age of 10 years. Children diagnosed before the age of 10 years exhibited significant weight gain after induction therapy, which persisted at the end of treatment. Conversely, those diagnosed at the age of 10 years or over had a non-significant decrease in BMI z-score after induction and recovered weight after the end of therapy (Fig. 2E).

We used a multivariable regression model to investigate the predictors of BMI z-score postinduction therapy and at the end of treatment. We found that gender, age at diagnosis, BMI z-score at diagnosis, WBC count at diagnosis, risk stratification, and chemotherapy protocol explained 55.2% of the variation in postinduction BMI z-score. Age and BMI z-score at diagnosis and the chemotherapy protocol were significant predictors of postinduction BMI z-score. For each 1-month increase of the age at diagnosis and 1 unit increase in the BMI z-score at diagnosis, the postinduction BMI z-score decreased by 0.008 and increased by 0.706, respectively. Treatment with the BFM-95 protocol was associated with a decrease in postinduction BMI z-score by 0.416 (Table 2). The same variables explained 36.6% of the variation in BMI z-score at the end of therapy. The only significant predictor of BMI z-score at this time point was BMI z-score at diagnosis, such that for each 1 unit increase in BMI z-score at diagnosis, end of therapy BMI z-score increased by 0.374 (Table 2).

**Table 2:** Association between demographic and clinical factors and BMI z-score after induction therapy (1 month) and at the end of treatment (24 months).

	Post-induction BMI z-score (n = 208)		Post-treatment BMI z-score (n = 135)	
	β (95% CI)	p-value	β (95% CI)	p-value
Gender				
Female	Ref		Ref	
Male	0.127 (− 0.145; 0.399)	0.357	− 0.185 (− 0.526; 0.155)	0.283
Age at diagnosis, months	− 0.008 (− 0.011; 0.005)	< 0.001	− 0.003 (− 0.007; 0.001)	0.209
Chemotherapy protocol				
GBTLI	Ref		Ref	
BFM-95	− 0.416 (− 0.763; − 0.069)	0.019	− 0.151 (− 0.633; 0.331)	0.536
WBC count at diagnosis	− 0.001 (− 0.002; 0.001)	0.437	0.000 (− 0.001; 0.002)	0.547
Risk stratification				
Low risk	Ref		Ref	
Medium risk	− 0.103 (− 0.532; 0.326)	0.636	0.415 (− 0.214; 1.044)	0.194
High risk	0.117 (− 0.274; 0.508)	0,557	− 0.173 (− 0.674; 0.329)	0.497
BMI z-score at diagnosis	0.706 (0.594; 0.819)	< 0.001	0.374 (− 0.007; 0.001)	< 0.001

*95%CI* 95% confidence interval, *BFM* European Group Berlin-Frankfurt-Munich protocol, *BMI* body mass index, *GBTLI* Brazilian Group for the Treatment of Childhood Leukemia, *WBC* white blood cell.

### Association between nutritional status, disease relapse, and overall survival

We also examined the association between nutritional status and prognostic measures among 120 children who died or completed follow-up until 4 years after initial treatment. Univariate analysis indicated that none of the assessed demographic, clinical, and treatment-related variables were associated with higher risk of relapse at the 4-year follow-up (Table 3). Multiple Cox regression analyses including the covariates age at diagnosis, CNS disease, and WBC count at diagnosis, indicated that weight status assessed by BMI was not associated with the risk ALL relapse at 4 years (Table 3). However, the age of 5 years or over at diagnosis (versus less than 5 years) and CNS disease increased by 2.23 and 6.50 times, respectively, the risk of ALL relapse at 4 years (Table 3).

**Table 3:** Multivariate logistic regression for overall survival and ALL relapse at 4-year follow-up (n = 120).

	Overall survival				ALL relapse			
	Univariate analysis		Multivariate analysis*		Univariate analysis		Multivariate analysis*	
	HR (IC 95%)	p-value	HR (IC 95%)	p-value	HR (IC 95%	p-value	HR (IC 95%)	p-value
Gender		0.8743						
Female	1	–			1			
Male	1.06 (0.52; 2.14)	0.8743			1.09 (0.53; 2.27)	0.8126		
Age								
< 5 years	1		1		1		1	
≥ 5 years	2.63 (1.21; 5.72)	0.0145	2.24 (1.00; 5.00)	0.0486	2.01 (0.95; 4.28)	0.0688	2.23 (1.00; 5.02)	0.0426
Chemotherapy protocol								
BFM-95	2.47 (1.16; 5.26)	**0.0184**	2.11 (1.00; 4.55)	0.0461	1.48 (0.70; 3.12)	0.3003		
GBTLI	1	–	1		1			
Risk stratification								
High	2.06 (0.70; 6.04)	0.1859			2.17 (0.73; 6.45)	0.1618		
Low	1	–			1			
Standard	1.81 (0.53; 6.18)	0.3447			2.16 (0.62; 7.53)	0.2252		
WBC count at diagnosis (× 10^9^/L)		0.0759						
< 10,000	1	–			1			
10,000 to 49,999	2.58 (1.15; 5.77)	**0.0208**			2.02 (0.82; 4.99)	0.1259		
50,000 to 99,999	0.87 (0.19; 3.94)	0.6809			2.50 (0.85; 7.33)	0.0939		
≥ 100,000	2.43 (0.84; 7.02)	0.0992			2.93 (1.00; 8.57)	0.0502		
CNS disease								
Negative	1	–			1			
Positive	2.57 (0.61; 10.80)	0.1962			4.16 (0.99; 17.61)	0.0525	6.50 (1.42; 29.71)	0.0159
BMI category								
Underweight	1.13 (0.27; 4.76)	0.8713	1.03 (0.24; 4.36)	0.9672	1.13 (0.26; 4.80)	0.8698	1.09 (0.25; 4.70)	0.9035
Normal weight	1		1		1		1	
Overweight/obesity	1.44 (0.50; 4.14)	0.4987	1.09 (0.37; 3.20)	0.8730	1.62 (0.56; 4.71)	0.3706	1.31 (0.44; 3.93)	0.6293

*95%CI* 95% confidence interval, *BFM* European Group Berlin–Frankfurt–Munich protocol, *BMI* body mass index, *GBTLI* Brazilian Group for the Treatment of Childhood Leukemia, *HR* hazard ratio, *WBC* white blood cell. Significant values are in bold.

*Multivariate analysis including the covariates sex, age, disease risk stratification (low, standard, high), chemotherapy protocol (BFM, GBTLI), WBC (100,000), and CNS disease (negative, positive).

Univariate analyses indicated that age of 5 years or over at diagnosis (versus less than 5 years), treatment with BFM-95 (versus GBTLI protocol), and WBC count at diagnosis between 10,000 and 49,000 (versus < 10,000 × 10^9^/L) were significantly associated with the risk of poorer overall survival at 4 years follow-up (Table 3). Multiple Cox regression analyses including the covariates age at diagnosis, chemotherapy protocol, CNS disease, and WBC count at diagnosis, indicated that weight status assessed by BMI was not associated with the risk of poorer overall survival at 4 years (Table 3). However, age of 5 years or over at diagnosis (versus less than 5 years) and treatment with BFM-95 (versus GBTLI protocol) increased by 2.24 and 2.11 times, respectively, the risk of poorer overall survival at 4 years (Table 3).

## Discussion

In this cohort of children with ALL, we examined body mass trajectory from diagnosis to the completion of treatment. We found that weight gain was frequent and occurred early in ALL therapy, with almost 20% of children being overweight or obese at the end of treatment. Moreover, BMI z-score following induction therapy was predicted by age at diagnosis, the type of chemotherapy protocol, and BMI at diagnosis, whereas BMI z-score at the end of treatment was predicted by BMI z-score at diagnosis. Despite the association between BMI at diagnosis and increased BMI throughout treatment, we found no association between the nutritional status at diagnosis and clinical outcomes. These findings have potential clinical implications, implying that weight management strategies should be implemented early during ALL treatment.

Our findings are consistent with those from previous studies addressing weight trends of children with ALL during and after treatment^16,21,22^. We found that weight gain occurred most prominently following induction therapy and weight remained stable until treatment completion. However, our findings indicated that children aged 10 years or more at diagnosis did not exhibit the overall trend for weight gain throughout treatment, the incidence of overweight/obesity at the diagnosis of pediatric ALL was lower than that reported in most previous studies, and weight status was not associated with relapse or overall survival at 4 years.

Weight gain in the setting of ALL treatment is attributed to several factors, particularly prolonged exposure to glucocorticoids and unhealthy habits. Glucocorticoids affect many aspects of energy homeostasis and are well-known to increase energy intake, stimulate fat storage by inducing differentiation of preadipocytes to adipocytes, and promote adipose tissue hypertrophy^16,23–25^. Other chemotherapeutic agents, such as anthracyclines and vincristine, may also contribute to changes in nutrition status by affecting energy expenditure through their influences on cardiovascular or muscle strength^26,27^.

In addition to the effects of chemotherapeutic agents, it is not uncommon for children with ALL to have unhealthy eating and activity behaviors^28^. Due to the impact of ALL diagnosis and cancer treatment, parents tend to be more permissive on nutritional and physical activity habits throughout therapy^28,29^. Despite perceiving unhealthy lifestyle behaviors^30^, parents experience difficulties improving dietary habits and reversing physical inactivity following ALL therapy completion^30^. This may further contribute to weight gain and adversely impact long-term outcomes.

Body mass trajectory in pediatric ALL survivors has been assessed in some studies following more extended periods after completion of therapy. In a meta-analysis of 47 studies involving 1742 pediatric ALL survivors, the BMI z-score percentile was higher than that of a reference population or a control group. Moreover, although the prevalence of overweight and obesity was higher after a shorter time since the completion of therapy, it was still high in studies examining survivors 10 or more years after treatment^22^. Some studies have also indicated that obesity following pediatric ALL therapy is associated with an increased prevalence of cardiovascular risk factors^31^. In the French ‘Leukemia in Children and Adolescents’ cohort, comprising 1025 adult survivors of pediatric ALL, with a mean follow up of 16.2 years, metabolic syndrome was reported in 10.3% of patients, compared with 4.5% of a control population^32^. Taken together, these findings indicate that it is important to examine body mass trajectories during and following ALL treatment to identify the sensitive windows of unhealthy weight gain and the predictors of weight increase, since this could aid in the development of strategies to prevent treatment-related obesity and its long-term consequences among ALL survivors.

We found that BMI z-score at the time of ALL diagnosis was the most important predictor of BMI z-score after induction therapy and at the end of treatment. The same result was observed by Zhang et al.^18^, who reported that patients who were overweight or obese at the diagnosis of ALL were 11.9 times more likely to be overweight/obese at the end of treatment than those who were underweight or had healthy weight at the diagnosis. Similar results were observed in other studies involving children with ALL^33,34^, reinforcing the body mass changes observed herein.

We found that children aged 2 to 10 years at the diagnosis of ALL had higher BMI z-scores than patients aged 10 years or over. Moreover, younger children gained significantly more weight in the induction phase, but their BMI z-scores remained stable from induction to the completion of therapy. Conversely, older exhibited a reduction in their BMI z-scores at induction, which was recovered at the end of treatment and remained significantly lower when compared with younger children. The association between age at ALL diagnosis and increased weight gain or obesity during therapy is inconsistent when comparing data from previous studies.

Similarly to our findings, some studies reported that younger children exhibit higher weight increases during ALL therapy^34–37^. Others found no association between age at diagnosis and BMI z-score changes throughout treatment^38–41^. A meta-analysis including 1742 pediatric ALL survivors indicated weight gain was frequent following treatment but was not associated with younger age at diagnosis or other features such as gender and cranial irradiation^22^. Notably, in the latter meta-analysis, the authors reported that the method of defining young age at diagnosis varied among studies^22^, limiting the interpretation of the findings. Therefore, future studies are needed to determine the association between age at diagnosis and body mass trends during and after ALL treatment and whether behavioral or biological features may account for the difference between older and younger children.

Children included in the present study were treated with the GBTLI or the BFM-95 protocol. The latter involves the administration of methotrexate at higher doses when compared with GBLTI. Because of these differences, we hypothesized that the GBTLI and BFM-95 protocols could affect body mass trajectories differently. After completion of treatment, children undergoing either protocol exhibited a substantial increase in BMI z-score compared with BMI z-score at diagnosis. In agreement with our findings, data from recent studies reported no differences in weight gain at the end of therapy in patients undergoing different chemotherapy protocols^42,43^. However, the increase in BMI z-score of lesser magnitude in children undergoing the BFM-95 protocol compared to those treated with the GBTLI protocol, although not statistically significant, may be explained by the fact that the first involves the administration of methotrexate more intensively, at higher doses, following the induction phase^44,45^. Notably, children treated with the GBTLI protocol had a higher BMI z-score at diagnosis when compared with those treated with the BFM-95 protocol, although the difference was not statistically significant.

The higher toxicity of the BFM-95 protocol may also explain the higher risk of death we observed in children treated with the latter protocol compared to the GBTLI protocol. Children aged 10 years or over also had a higher risk of death when compared to younger children. This agrees with data from previous studies reporting poorer ALL outcomes among children who are older compared with those who are aged 1 to 10 years at the time of diagnosis. This most likely results from several factors, such as more unfavorable cytogenetic features, more frequent presentation as a higher-risk disease among older children, and more frequent treatment-related complications in the older age group^46^.

The association between nutritional status at diagnosis and outcomes in pediatric ALL has been addressed in several studies, and meta-analysis findings indicate that children who are overweight or obese at the time of diagnosis exhibit worse outcomes, such as reduced event-free survival and overall survival^11,13^. However, it is noteworthy that other studies also failed to support that nutritional status at diagnosis was associated with poorer event-free survival and overall survival in children with ALL, similarly to our findings^47–49^.

The reasons for the divergent findings between our findings and those from most other studies reporting a negative impact of overweight or obesity on childhood ALL outcomes are not clear. The lack of association in our analyses could be attributed, in part, to the relatively small number of children with complete data at the 4-year follow-up. Another important point to be highlighted is that we found a relatively low rate of excess body weight among children with ALL. Despite being consistent with the prevalence of overweight in the Brazilian pediatric population^50^, the percentage of overweight and obese children is lower than that found in studies carried out in other countries^11^ and may have contributed to the failure to identify an association between being overweight or obese at the time of ALL diagnosis and disease outcomes. Moreover, it is not possible to rule out that ethnic differences affecting biological and behavioral features may have accounted for the divergent findings.

Despite the divergent findings between different studies addressing the association between weight status at the diagnosis of ALL and its prognosis, it should be pointed out that this association is biologically plausible. There is evidence from animal models and in vitro studies supporting that excess adiposity is mechanistically linked to worse pediatric ALL prognosis^51–53^, as has been thoroughly reviewed elsewhere^54^, by several mechanisms, such as increased fuel availability (free fatty acids)^55^, the attraction of leukemia cells to adipose tissue and their protection from chemotherapeutic drugs^52^, and secretion of cytokines and growth factors that favor their proliferation and survival^54^.

This study has some limitations that should be considered interpreting of our findings, and that should be addressed in future studies. First, we relied on BMI z-score to measure of overweight and obesity. Given that it is not overall body mass but adiposity which most likely influences ALL outcomes, it would be essential to assess the trajectory of body fat during and after treatment to gain more reliable insights into the changes in body fat throughout therapy and even the relationship between body composition and ALL prognosis. Second, we did not consider the influence of some variables that may affect body weight, including genetics, diet, physical activity, and psychological factors. The latter factors are important to improve our understanding of the factors contributing to different weight status trends during ALL therapy and in ALL survivors. Also, we could not examine weight status after a longer follow-up following the completion of treatment to determine whether BMI z-score gain during therapy persists in the long term.

Our results confirm those from previous studies indicating the increase in body weight during treatment of pediatric ALL and have important implications for the care of children and adolescents with the disease. Given the advances in ALL therapy, approximately 90% of children and adolescents treated for the disease will be cured^19^. The increasing population of long-term ALL survivors faces a high rate of various problems, including obesity, which substantially affects morbidity and mortality^9^. The finding that BMI z-score increases early during ALL therapy and that the BMI z-score at the diagnosis was the most important predictor of BMI z-score at the end of the induction phase and after therapy completion strongly suggests that the nutritional status should be assessed at all stages of treatment and that weight management interventions should be implemented early for children who are overweight or obese at diagnosis.

## Methods

### Subjects

This was a retrospective cohort study conducted according to the STROBE guidelines^56^. We used secondary data from patients aged 1 to 18 years and diagnosed with ALL, who were followed up at the Children’s Hospital of Brasilia, Brazil, between January 2012 and July 2020. We excluded patients with infant leukemia, acute leukemia of ambiguous lineage, Philadelphia chromosome-positive leukemia, T-cell ALL, Down’s syndrome, and chronic non-progressive encephalopathy. Those without weight and height data at ALL diagnosis, that dropped out of treatment, died before the first induction therapy, or were transferred to another hospital were also excluded. The study was approved by the Ethics Review Committee from the School of Health Sciences of the University of Brasilia, and informed consent was waived by the latter institution (protocol number 04495418.9.0000.5553). All methods and procedures were performed in accordance with the Declaration of Helsinki.

### Data collection

Medical records were assessed for information on age at ALL diagnosis, gender, initial WBC, CNS disease, and chemotherapy treatment protocol. Anthropometric data (weight and height) were collected at three time points: before treatment, after induction therapy, and at the end of treatment. We also collected information related to prognosis, including ALL relapse and overall survival (OS) at 48-month follow-up. OS was determined as the time from diagnosis to death from any cause. Patients who did not experience any event were censored at the date of last clinical contact.

Patients were treated with the BFM-95 protocol^45^ or the GBTLI protocol^44^ (Supplementary Material). For both protocols, clinical and laboratory characteristics are employed to stratify patients into standard, intermediate, and high-risk of relapse. Both protocols were used in the Children’s Hospital of Brasilia to treat pediatric ALL, and the researchers had no interference with assigning study subjects to either protocol.

For data analysis, nutritional status was categorized into underweight, normal weight (risk of overweight and eutrophic), and overweight (overweight and obesity). WHO Child Growth Standards was used to classify the nutritional status of children aged 5 years or less, and the WHO growth reference was used for school-aged children and adolescents. Overweight was defined as a BMI above one standard deviation and obesity as a BMI above two standard deviations of the *z*-score for age and sex^57,58^.

### Statistical analyses

Continuous variables were presented as the median and interquartile range (IQR), as most of them were non-normally distributed, and categorical variables were presented as frequencies. Demographic and clinical data from non-overweight/obese and overweight/obese children were compared using Mann–Whitney, Fisher’s exact, or chi-squared tests. BMI-for-age z-score at diagnosis, post-induction therapy, and end of treatment were compared by analysis of variance followed by Tukey’s multiple comparison test. BMI-for-age z-score at each time point was compared according to gender, age at ALL diagnosis, and chemotherapy regimen using the unpaired t-test. The normality of data was assessed using the Shapiro–Wilk test.

Multiple linear regression analysis was used to assess the association between demographic and clinical variables and BMI z-score at 1 month (end of induction) and 2 years (end of treatment) after ALL diagnosis.

To examine the impact of weight category and other predictors on ALL relapse and OS, we used adjusted Cox regression models for time to disease relapse or death occurrence associated with BMI category at diagnosis (underweight, normal weight, or overweight/obese) adjusted for demographic and clinical covariates, using risk ratio as the measure of effect. Time (in months) for the occurrence of disease relapse or death were considered the dependent variable. BMI category was considered the independent variable, and the covariates selected as possible confounders were sex, age, disease risk stratification (low, standard, high), chemotherapy protocol (BFM-95, GBTLI), WBC (< 10,000; 10,000 to 49,000; 50,000 to 99,999, > 100,000), and CNS disease (negative, positive). The analysis was conducted in two stages. First, simple Cox regression models were fitted for each covariate and those associated with the dependent variable with a significance level < 0.25 were included in the multiple Cox regression analysis. Subsequently, adjustments were made for these variables through a process of their inclusion and removal, and those associated with the dependent variable with a significance level < 0.05 remained in the final model. Finally, the BMI category (independent variable of interest) was included to verify its degree of association with time to relapse or time to death after adjusting for possible confounders. Hazard ratios (HR) and their corresponding 95% confidence intervals (95% CI) were calculated.

Multicollinearity between independent variables was assessed. A tolerance indicator > 0.60 was considered as the limit for the presence of multicollinearity. Significance was considered at p values < 0.05. The analyzes were conducted using the SAS software, version 9.4.

### Supplementary Information

Supplementary Information.

## Data Availability

**A**ll data was presented in the manuscript. Individual deidentified participant data will be provided upon request (paulagalati@gmail.com or angelicamato@unb.br).
