# Supplementary material for: Body mass trajectory from diagnosis to the end of treatment in a pediatric acute lymphoblastic leukemia cohort
Source: Sci Rep. 2023 Aug 21;13:13590. doi: 10.1038/s41598-023-39287-z (PMC10442422; doi:10.1038/s41598-023-39287-z)
Supplement: Supplementary file 1 — Supplementary Information. [file 41598_2023_39287_MOESM1_ESM.docx]

**Supplementary Data**

**Acute Lymphoblastic Leukemia Berlin -Frankfurt-Münster treatment protocol (BFM-95 protocol) ^1^.**

| **Standard risk** | **Dose** | **Days of administration** |
| --- | --- | --- |
| *Induction/consolidation* |  |  |
| *Phase A (33 days)* |  |  |
| Prednisone (PO) | 60 mg/m^2^/day | 1-28 |
| Vincristine (IV) | 1.5 mg/m^2^/dose | 8, 15, 22, 29 |
| Daunorubicine (PI) | 30 mg/m^2^/dose | 8, 15 |
| L-asparaginase (PI) | 5000 IU/m^2^/dose | 12, 15, 18, 21, 24, 27, 30, 33 |
| Methotrexate (IT) | 12 mg/dose ^a^ | 1, 12, 33 |
| *Phase B (28 days)* |  |  |
| Cyclophosphamide (PI) | 1000 mg/m^2^/dose | 36, 64 |
| Cytarabine (IV) | 75 mg/m^2^/dose | 38-41, 45-48, 52-55, 59-62 |
| 6-mercaptopurine (PO) | 60 mg/m^2^/day | 36-63 |
| Methotrexate (IT) | 12 mg/dose ^a^ | 45, 59 |
| *Extracompartment therapy (56 days)* |  |  |
| 6-mercaptopurine (PO) | 25 mg/m^2^/day | 1-56 |
| Methotrexate (PI) | 5000 mg/m^2^/dose | 8, 22, 36, 50 |
| Methotrexate (IT) | 12 mg/dose ^a^ | 8, 22, 36, 50 |
| Cytarabine (PI) | 200 mg/m^2^/dose | 9, 23, 37, 51 |
| *Reinduction* |  |  |
| *Phase A (28 days)* |  |  |
| Dexamethasone (PO) | 10 mg/m^2^/day | 1-21 |
| Vincristine (IV) | 1.5 mg/m^2^/dose | 8, 15, 22, 29 |
| Doxorubicin (PI) | 30 mg/m^2^/dose | 8, 15, 22, 29 |
| L-asparaginase (PI) | 10000 IU/m^2^/dose | 8, 11, 15, 18 |
| *Phase B (14 days)* |  |  |
| Cyclophosphamide (PI) | 1000 mg/m^2^/dose | 36 |
| Cytarabine (IV) | 75 mg/m^2^/dose | 38-41, 45-48 |
| 6-thioguanine (PO) | 60 mg/m^2^/dose | 36-49 |
| Methotrexate (IT) | 12 mg/dose ^a^ | 45, 59 ^c^ |
| *Maintenance therapy* |  |  |
| Methotrexate (PO) | 20 mg/m^2^/week ^b^ | Until 104 weeks (girls) or 156 weeks (boys) from initial diagnosis |
| 6-mercaptopurine (PO) | 50 mg/m^2^/day ^b^ | Until 104 weeks (girls) or 156 weeks (boys) from initial diagnosis |
| **Medium risk** | **Dose** | **Days of administration** |
| *Induction/consolidation* |  |  |
| *Phase A (33 days)* |  |  |
| Prednisone (PO) | 60 mg/m^2^/day | 1-28 |
| Vincristine (IV) | 1.5 mg/m^2^/dose | 8, 15, 22, 29 |
| Daunorubicine (PI) | 30 mg/m^2^/dose | 8, 15, 22, 29 |
| L-asparaginase (PI) | 5000 IU/m^2^/dose | 12, 15, 18, 21, 24, 27, 30, 33 |
| Methotrexate (IT) | 12 mg/dose ^a^ | 1, 12, 33 |
| *Phase B (28 days)* |  |  |
| Cyclophosphamide (PI) | 1000 mg/m^2^/dose | 36, 64 |
| Cytarabine (IV) | 75 mg/m^2^/dose | 38-41, 45-48, 52-55, 59-62 |
| 6-mercaptopurine (PO) | 60 mg/m^2^/day | 36-63 |
| Methotrexate (IT) | 12 mg/dose ^a^ | 45, 59 |
| *Extracompartment therapy (56 days)* |  |  |
| 6-mercaptopurine (PO) | 25 mg/m^2^/day | 1-56 |
| Methotrexate (PI) | 5000 mg/m^2^/dose | 8, 22, 36, 50 |
| Methotrexate (IT) | 12 mg/dose ^a^ | 8, 22, 36, 50 |
| Cytarabine (PI) | 200 mg/m^2^/dose | 9, 23, 37, 51 |
| *Reinduction* |  |  |
| *Phase A (28 days)* |  |  |
| Dexamethasone (PO) | 10 mg/m^2^/day | 1-21 |
| Vincristine (IV) | 1.5 mg/m^2^/dose | 8, 15, 22, 29 |
| Doxorubicin (PI) | 30 mg/m^2^/dose | 8, 15, 22, 29 |
| L-asparaginase (PI) | 10000 IU/m^2^/dose | 8, 11, 15, 18 |
| *Phase B (14 days)* |  |  |
| Cyclophosphamide (PI) | 1000 mg/m^2^/dose | 36 |
| Cytarabine (IV) | 75 mg/m^2^/dose | 38-41, 45-48 |
| 6-thioguanine (PO) | 60 mg/m^2^/dose | 36-49 |
| Methotrexate (IT) | 12 mg/dose ^a^ | 45, 59 ^c^ |
| *Maintenance therapy* |  |  |
| Methotrexate (PO) | 20 mg/m^2^/week ^b^ | Until 104 weeks from initial diagnosis |
| 6-mercaptopurine (PO) | 50 mg/m^2^/day ^b^ | Until 104 weeks from initial diagnosis |
| **High risk** | **Dose** | **Days of administration** |
| *Induction/consolidation* |  |  |
| *Phase A (29 days)* |  |  |
| Prednisone (PO) | 60 mg/m^2^/day | 1-21 |
| Vincristine (IV) | 1.5 mg/m^2^/dose | 8, 15, 22, 29 |
| Daunorubicine (PI) | 30 mg/m^2^/dose | 8, 15, 22, 29 |
| L-asparaginase (PI) | 5000 IU/m^2^/dose | 12, 15, 18, 21, 24, 27 |
| Methotrexate (IT) | 12 mg/dose ^a^ | 1, 12, 27 |
| *Phase B (28 days)* |  |  |
| Cyclophosphamide (PI) | 1000 mg/m^2^/dose | 36, 64 |
| Cytarabine (IV) | 75 mg/m^2^/dose | 38-41, 45-48, 52-55, 59-62 |
| 6-mercaptopurine (PO) | 60 mg/m^2^/day | 36-63 |
| Methotrexate (IT) | 12 mg/dose ^a^ | 45, 59 |
| *Intensified consolidation/extracompartment therapy (56 days), x 2* |  |  |
| Element HR-1 |  |  |
| Dexamethasone (PO) | 20 mg/m^2^/day | 1-5 |
| Vincristine (IV) | 1.5 mg/m^2^ | 1^d^, 6^d^ |
| Methotrexate (PI) | 5000 mg/m^2^/dose | 1 |
| Cyclophosphamide (PI) | 200 mg/m^2^/dose | 2-4 |
| Cytarabine (PI) | 2 g/m^2^/dose | 5 |
| L-asparaginase (PI) | 25000 IU/m^2^/dose | 6 |
| Methotrexate/cytarabine/prednisolone (IT) | 12/30/10 mg/dose^a^ | 1 |
| Element HR-2 |  |  |
| Dexamethasone (PO) | 20 mg/m^2^/day | 1-5 |
| Vindesine (IV) | 3 mg/m^2^/dose | 1,6 |
| Methotrexate (PI) | 5000 mg/m^2^/dose | 1 |
| Iphosphamide (PI) | 800 mg/m^2^/dose | 2-4 |
| Daunorubicin (PI) | 30 mg/m^2^/dose | 5 |
| L-asparaginase (PI) | 25000 IU/m^2^/dose | 6 |
| Methotrexate/cytarabine/prednisolone (IT) | 12/30/10 mg/dose^a^ | 1 ^e^ |
| Element HR-3 |  |  |
| Dexamethasone (PO | 20 mg/m^2^/day | 1-5 |
| Cytarabine (PI) | 2 g/m^2^/dose | 1-2 |
| Etoposide (PI) | 100 mg/m^2^/dose | 3-5 |
| L-asparaginase (PI) | 25000 IU/m^2^/dose | 6 |
| Methotrexate/cytarabine/prednisolone (IT) | 12/30/10 mg/dose^a^ | 5 |
| *Reinduction* |  |  |
| *Phase A (28 days)* |  |  |
| Dexamethasone (PO) | 10 mg/m^2^/day | 1-21 |
| Vincristine (IV) | 1.5 mg/m^2^/dose | 8, 15, 22, 29 |
| Doxorubicin (PI) | 30 mg/m^2^/dose | 8, 15, 22, 29 |
| L-asparaginase (PI) | 10000 IU/m^2^/dose | 8, 11, 15, 18 |
| *Phase B (14 days)* |  |  |
| Cyclophosphamide (PI) | 1000 mg/m^2^/dose | 36 |
| Cytarabine (IV) | 75 mg/m^2^/dose | 38-41, 45-48 |
| 6-thioguanine (PO) | 60 mg/m^2^/dose | 36-49 |
| Methotrexate (IT) | 12 mg/dose ^a^ | 45, 59 ^c^ |
| *Maintenance therapy* |  |  |
| Methotrexate (PO) | 20 mg/m^2^/week ^b^ | Until 104 weeks from initial diagnosis |
| 6-mercaptopurine (PO) | 50 mg/m^2^/day ^b^ | Until 104 weeks from initial diagnosis |

^a^ Doses adjusted for children younger than 3 years.

^b^ Doses adjusted to white blood cell count (target range, 2000-3000/uL).

^c^ Children with central nervous system status CNS 3 received additional IT methotrexate on days 1 and 18.

^d^ Vincristine was omitted in the first HR-1 course.

^e^ Children with central nervous system status CNS 3 received additional IT methotrexate on day 5.

PO: orally; IV: intravenous push; PI: intravenous infusion; IT: intrathecally

**Brazilian Group for the Treatment of Childhood Leukemia treatment protocol (GBTLI protocol) ^2^.**

| **Standard/medium risk ^a^** | **Dose** | **Days of administration** |
| --- | --- | --- |
| *Induction/consolidation* |  |  |
| Prednisone (PO) | 60 mg/m^2^ | 1-28 |
| Vincristine (IV) | 1.5 mg/m^2^ | 8, 15, 22, 29 |
| Daunorubicine (PI) | 30 mg/m^2^ | 8, 15, 22, 29 |
| L-asparaginase (PI) | 5000 IU/m^2^ | 12, 15, 18, 21, 24, 27, 30, 33 |
| Methotrexate (IT) | 12 mg/dose ^b^ | 1, 12, 33, 45, 49 (45 and 59 if CNS disease) |
| *Protocol M (56 days)* |  |  |
| 6-mercaptopurine (PO) | 25 mg/m^2^ | 1-56 |
| Methotrexate (PI) | 2000 mg/m^2^ | 8, 22, 36, 50 |
| Methotrexate (IT) | 6-12 mg | 8, 22, 36, 50 |
| *Protocol II (49 days)* |  |  |
| Dexamethasone (PO) | 10 mg/m^2^ | 1-22 |
| Vincristine (IV) | 1.5 mg/m^2^ | 8, 15, 22, 29 |
| Doxorubicin (IV) | 30 mg/m^2^ | 8, 15, 22, 29 |
| L-asparaginase (IM) | 10000 IU m^2^ | 8, 11, 15, 18 |
| Cyclophosphamide (IV) | 1000 mg/m^2^ | 36 |
| 6-thyoguanine (PO) | 60 mg/m^2^ | 36-49 |
| Cytarabine (IV) | 75 mg/m^2^ | 38-41, 45-48 |
| Methotrexate (IT) | 12 mg ^b^ | 38-45 |
| *Maintenance* |  |  |
| 6-mercaptopurine (PO) | 50 mg/m^2^ | Until 104 weeks from initial diagnosis |
| Methotrexate (PO) | 50 mg/m^2^/week | Until 104 weeks from initial diagnosis |
| **High risk ^a^** | **Dose** | **Days of administration** |
| *Induction/consolidation* |  |  |
| Prednisone (PO) | 60 mg/m^2^ | 1-28 |
| Vincristine (IV) | 1.5 mg/m^2^ | 8, 15, 22, 29 |
| Daunorubicine (PI) | 30 mg/m^2^ | 8, 15, 22, 29 |
| L-asparaginase (PI) | 5000 IU/m^2^/ | 12, 15, 18, 21, 24, 27, 30, 33 |
| Methotrexate (IT) | 12 mg ^b^ | 1, 12, 33, 45, 49 (45 and 59 if CNS disease) |
| *Intensified consolidation, x 2* |  |  |
| HR1 |  |  |
| Methotrexate (PI) | 2000 mg/m^2^ | 1 |
| Cyclophosphamide (IV) | 200 mg/m^2^ | 2-4 |
| Vincristine (IV) | 1.5 mg/m^2^ | 1 |
| Dexamethasone (PO) | 20 mg/m^2^ | 1-5 |
| Cytarabine (IV) | 2000 mg/m^2^ | 5 (every 12 h) |
| Methotrexate/cytarabine/dexamethasone (IT) | 6-12 mg / 16-30 mg / 2 mg/m^2^ | 1 |
| HR2 |  |  |
| Dexamethasone (PO) | 20 mg/m^2^ | 1-5 |
| Vincristine (IV) | 1.5 mg/m^2^ | 1 |
| Daunorubicine (IV) | 30 mg/m^2^ | 1 |
| Methotrexate (IV) | 2000 mg/m^2^ | 1 |
| Iphosphamide (IV) | 800 mg/m^2^ | 2-4 (very 12 h) |
| L-asparaginase (IM) | 10000 IU m^2^ | 6 |
| Methotrexate/cytarabine/dexamethasone (IT) | 6-12 mg / 16-30 mg / 2 mg/m^2^ | 5 |
| HR3 |  |  |
| Dexamethasone (PO) | 20 mg/m^2^ | 1-5 |
| Cytarabine (IV) | 2000 mg/m^2^ | 1-2 (every 12 h) |
| Etoposide (IV) | 100 mg/m^2^ | 3-5 (every 12 h) |
| L-asparaginase (IM) | 10000 IU m^2^ | 6 |
| Methotrexate/cytarabine/dexamethasone (IT) | 6-12 mg / 16-30 mg / 2 mg/m^2^ | 5 |
| *Protocol II (49 days)* |  |  |
| Dexamethasone (PO) | 10 mg/m^2^ | 1-22 |
| Vincristine (IV) | 1.5 mg/m^2^ | 8, 15, 22, 29 |
| Doxorubicin (IV) | 30 mg/m^2^ | 8, 15, 22, 29 |
| L-asparaginase (IM) | 10000 IU m^2^ | 8, 11, 15, 18 |
| Cyclophosphamide (IV) | 1000 mg/m^2^ | 36 |
| 6-thyoguanine (PO) | 60 mg/m^2^ | 36-49 |
| Cytarabine (IV) | 75 mg/m^2^ | 38-41, 45-48 |
| Methotrexate (IT) | 12 mg/dose ^b^ | 38-45 |
| *Maintenance* |  |  |
| 6-mercaptopurine (PO) | 50 mg/m^2^ | Until 104 weeks from initial diagnosis |
| Methotrexate (PO) | 50 mg/m^2^/week | Until 104 weeks from initial diagnosis |

Brazilian Group for the Treatment of Childhood Leukemia treatment protocol (Grupo Brasileiro de Tratamento de Leucemia da Infância, GBTLI protocol)

^a^ Medium and high risk children aged over one year receive prophylatic cranial irradiation (12 Gy) following Protocol II.

^b^ Doses adjusted according to age.

PO: orally; IV: intravenous push; PI: intravenous infusion; IT: intrathecally; IM: intramuscular injection.

**References**

1. Möricke, A. et al. Risk-adjusted therapy of acute lymphoblastic leukemia can decrease treatment burden and improve survival: treatment results of 2169 unselected pediatric and adolescent patients enrolled in the trial ALL-BFM 95. Blood 111, 4477-4489 (2008).

2. (INCA)., B. M. d. S. I. N. d. C. J. A. G. d. S. Condutas do INCA. Leucemia Agudas na Infância e na Adolescência. Revista Brasileira de Cancerologia 47, 245-257 (2001).
